# Phylogenetic analysis of *Eimeria tenella* isolated from the litter of different chicken farms in Mymensingh, Bangladesh

**DOI:** 10.1002/vms3.799

**Published:** 2022-04-05

**Authors:** Mohammad Zahangir Alam, Anita Rani Dey, Sharmin Aqter Rony, Shahnaz Parvin, Shirin Akter

**Affiliations:** ^1^ Department of Parasitology Faculty of Veterinary Science Bangladesh Agricultural University Mymensingh Bangladesh

**Keywords:** Bangladesh, chicken, *Eimeria tenella*, ITS1, phylogenetic analysis

## Abstract

**Background:**

Eimeria tenella is the most pathogenic intracellular protozoan parasite of seven *Eimeria* species causing chicken coccidiosis around the world. This species is particularly responsible for caecal coccidiosis leading to serious morbidity–mortality and financial loss in poultry production.

**Methods:**

The present study explored the genetic diversity of *E. tenella*. Litter slurry was collected from 18 broiler farms located in Mymensingh district, Bangladesh. Litter samples were processed for oocyst isolation–identification using parasitological techniques followed by genomic DNA extraction from sporulated oocysts. For molecular analysis, the *i* *n* *t* *e* *r* *n* *a* *l* *t* *r* *a* *n* *s* *c* *r* *i* *b* *e* *d* *s* *p* *a* *c* *e* *r* *1* gene of *E. tenella* was amplified using species‐specific primers and sequenced. After editing and alignment, 263 bp sequences were used for analysis.

**Results:**

Genetic analysis showed seven distinct genotypes and detected six single nucleotide polymorphisms among the 18 *E. tenella* isolates. The nucleotide and genotype diversity were 0.00507 and 0.8235, respectively. A phylogenetic tree was constructed with 66 sequences (seven studied genotypes and 59 reference sequences from GenBank database). The neighbour‐joining tree represented that the studied *E. tenella* isolates were grouped with reference *E. tenella* isolates with strong nodal support (100%) and the nucleotide sequences of *E. tenella*, *E. necatrix*, *E. acervulina*, *E. brunetti*, *E. maxima*, *E. mitis* and *E. praecox* formed separate clusters without any geographical boundaries.

**Conclusions:**

This is the first study on the genetic analysis of *E. tenella* from Mymensingh district, Bangladesh. These findings will provide baseline data on the species conformation and genetic variations of *E*. *tenella*. Further extensive investigation will be needed to reveal the population genetic structure of this parasite and thus will facilitate the planning of effective control strategies.

## Graphical abstract

Neighbor joining phylogeny of 66 ITS1 gene sequences of E. tenella. The analysis involved 66 nucleotide sequences (7 studied genotype sequences and 59 reference sequences, retrived from GenBank database). The sequences were aligned and constructed a neighbor joining tree. The percentage of replicate trees in which the associated taxa clustered together in the bootstrap test (1000 replicates) are shown next to the branches. The tree is drawn to scale, with branch lengths in the same units as those of the evolutionary distances used to infer the phylogenetic tree. Red bullets indicate studied genotype sequences.

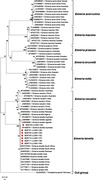

## 1 INTRODUCTION

Globally, the poultry industry is one of the fastest growing and vital agricultural sub‐sector in terms of food security and nutrition with an expected highest rise in demand for poultry now and in the near future (Mottet & Tempio, 2017). Unfortunately, poultry production is significantly threatened by a variety of pathogens including protozoan parasites. Among the protozoa ubiquitous, intracellular coccidian parasites of the genus *Eimeria* are the most economically important and most prevalent in chickens. Seven species of *Eimeria*, namely, *Eimeria tenella, E. necatrix, E. acervulina, E. maxima, E. brunetti, E. mitis* and *E. praecox* have been recorded from different parts of the chicken gastrointestinal tract (Soulsby, 1982). The species of *Eimeria* are involved in the repeated invasion and rupture of host intestinal epithelial cell, leading to enteritis and sometimes haemorrhage. In commercial poultry farms, ingestion of infective sporulated oocysts through feed, water and litter by the neighbouring hosts facilitates rapid transmission among birds (Conway & McKenzie, 2007) Among seven chicken *Eimeria* species, *E. tenella* ranks top for pathogenicity and is responsible particularly for caecal coccidiosis.

*Eimeria tenella* specifically infects and disrupts epithelial cells of the caecal crypts of Lieberkhün, resulting in varying degrees of haemorrhages depending on parasite infective dose size and host factors (age, genotype and pre‐exposure; Macdonald et al., 2017). This parasite can cause moderate to severe morbidity (weight loss or less weight gain, dehydration, diarrhoea, blood loss and mortality in severe cases (Allen & Fetterer, 2002; Conway & McKenzie, 2007). Chicken *Eimeria* infections can also complicate bacterial infections with *Clostridium perfringens* (contributing to necrotic enteritis) and *Salmonella enterica* serovar Enteritidis or Typhimurium (Macdonald et al., 2017). Thus, coccidiosis in poultry incurs financial losses in terms of low productivity, concurrent infections and expense arising from treatment and management. Coccidiosis has been reported in almost all farms in America, Europe and Asia by litter sample examination (Fornace et al., 2013), and the estimated global annual loss due to coccidiosis to the poultry industry is USD 3 billion (Jatau et al., 2014).

Chicken coccidiosis is considered economically crucial in commercial poultry operations in Bangladesh as well with an average 22%–34.48% bird‐level prevalence (Rony et al., 2021). Different chicken *Eimeria* species have been reported in different parts of Bangladesh using conventional and/or molecular tools. *Eimeria tenella* was found prevalent in all the study areas so far reported in Bangladesh (Alam et al., 2020, 2021; Karim & Begum, 1994; Karim & Trees, 1990). As *E. tenella* is the most deleterious chicken coccidian species and a well‐known model vaccine candidate for homogenous and heterogenous coccidian infection (Tang et al., 2018), accurate diagnosis and knowledge of the phylogenetic origin of this species are important. Oocyst morphology, sporulation time and location/scoring of pathological lesions were some clues for traditional diagnosis, which required specialist expertise and are prone to misinterpretation due to the high level of interspecies similarities of oocyst morphology (Long & Joyner, 1984). As genetic diversity influences epidemiology and pathogenicity, phylogenetic analysis of *E. tenella* could have a potential impact in developing diagnosis and a control strategy, generating knowledge on bionomics, epidemiology, immunogenomics and anticoccidial agent preference. Molecular techniques have been designated as reliable and specific tools to identify the species of *Eimeria* (Jenkins et al., 2006; Schnitzler et al., 1999). *I* *n* *t* *e* *r* *n* *a* *l* *t* *r* *a* *n* *s* *c* *r* *i* *b* *e* *d* *s* *p* *a* *c* *e* *r* (*I* *T* *S* *‐* *1*; Schnitzler et al., 1999), ITS‐2 (Lien et al., 2007) and the 5.8S ribosomal ribonucleic acid (*r* *R* *N* *A*; Molloy et al., 1998) genomic regions are targeted for the identification of species of *Eimeria*. Hence, the present study attempted to explore the diversity of different isolates of *E. tenella* from Bangladesh using the *I* *T* *S* *‐* *1* gene.

## 2 MATERIALS AND METHODS

### 2.1 Study area and selection of broiler farms

The study was carried out on 18 broiler farms of the Mymensingh district in Bangladesh. These farms were selected due to their ease of approachability and large flock size.

### 2.2 Collection of samples and isolation of oocysts

Approximately 250 g of litter sample slurry was collected from each farm area adjacent to feeder and water lines. Processing of litter samples and isolation–identification of oocysts were performed according to the protocol described by Alam et al. (2020). The floatation technique was applied to isolate and concentrate *Eimerian* oocysts using saturated sodium chloride (specific gravity 1.18–1.2) followed by microscopy. Sporulation of oocysts was triggered by culture at 28°C in 2.5% potassium dichromate for 1–2 days. Finally, the concentrated oocysts were pelleted using repeated centrifugation and elimination of media.

### 2.3 Extraction of genomic DNA

For genomic DNA extraction, pelleted sporulated oocysts were washed with a 1 mM sodium hypochlorite solution for 10 min at 4°C, followed by three‐times rinsing with deionised water. To crack the oocyst wall and release the sporozoites, a specialised tissue homogeniser was used. Genomic DNA was isolated according to the manufacturer's instructions using the QIAamp stool DNA isolation kit (Qiagen). The extracted DNA samples were measured to ascertain the concentration of DNA and kept at –20°C until further use.

### 2.4 Amplification and sequencing of the *ITS1* gene of *E. tenella*

*Eimeria tenella* species were identified by a single PCR assay using species‐specific primers targeting the *I* *T* *S* *‐* *1* as previously described by Schnitzler et al. (1999). Forward (EtF: 5`‐AATTTAGTCCATCGCAACCCT‐3`) and reverse (EtR: 5`‐CGAGCGCTCTGCATACGACA‐3`) species‐specific primer sequences were used in this study. Amplification of the *I* *T* *S*‐*1* sequences of genomic rDNA was carried out in 25 μl reaction volumes containing 2 μl of DNA template, 10 pmol of reverse and forward primers, 3.0 mM MgCl_2_, 2.0 μl 10 X PCR Buffer, 200 μM of each deoxyribose nucleotide triphosphate and 0.4 U Taq DNA polymerase. The thermal program of PCR was as follows: denaturation step at 95°C for 5 min, 35 cycles of denaturation at 95°C for 30 s, annealing at 58°C or 65°C for 30 s and extension at 72°C for 1 min and a final extension at 72°C for 3 min. The successfully amplified PCR products were visualised using 2% agarose gel to verify that they represented a single band. All positive samples were purified using Qiaquick kits (Qiagen) and direct cycle sequencing of PCR‐amplified fragments was performed using a commercially available automated sequencer.

### 2.5 Phylogenetic analysis

The sequences of *I* *T* *S* *‐* *1* were aligned using the program Clustal W within MEGA v.6.0 (Tamura et al., 2013). Intra‐population diversity parameters such as nucleotide diversity, genotype diversity and the average number of nucleotide differences were calculated using DnaSP version 5.1 (Rozas, 2009). Pairwise comparisons were performed with previously published sequences, and identities (%) were calculated using the program BioEdit (Hall, 1999). Phylogenetic analysis was performed using neighbour‐joining based on the Tamura–Nei model (Tamura et al., 2013). Confidence limits were assessed using the bootstrap procedure (1000 replicates) for neighbour‐joining trees, and other settings were obtained using the default values in MEGA v.6.0 (Tamura et al., 2013). A 50% cut‐off value was implemented for the consensus tree.

## 3 RESULTS

### 3.1 Species identification and genotyping of *E. tenella*

**FIGURE 1:**
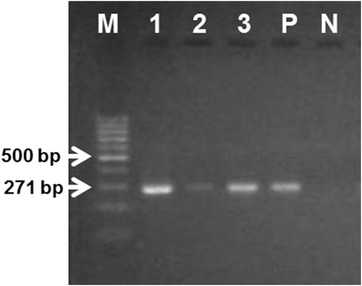
Agarose gel electrophoresis of the PCR products. Amplification of ∼271 bp *i* *n* *t* *e* *r* *n* *a* *l* *t* *r* *a* *n* *s* *c* *r* *i* *b* *e* *d* *s* *p* *a* *c* *e* *1* (*I* *T* *S* *1*) gene for *Eimeria tenella* represented on 1.5% agarose gel; M, 100 bp marker; 1–3, *I* *T* *S* *1* gene product from samples; P, positive PCR control; N, negative PCR control

A total of 18 *Eimeria* field samples were isolated from 18 local commercial broiler production farms in Mymensingh district. To confirm the species identity of *Eimeria* samples, species‐specific primers of *E. tenella* were used to amplify the *I* *T* *S* *1* region. The results revealed that all the field isolates were confirmed to be *E. tenella* since they represented a specific single 271 bp band using the specific primer of *E. tenella* (Figure 1). Positive PCR products of each isolate were subjected to sequencing, and sequences were edited, aligned and the resulting aligned 263 bp sequences were analysed. The sequences were compared with those in the NCBI database using the Basic Local Alignment Search Tool (BLAST) for nucleotide. Species identification was determined from the best‐scoring reference sequence of the BLAST output. Eighteen PCR positive samples that were sequenced for *E. tenella* showed 99.23%– 100% homology with *E. tenella* DNA sequences deposited in the GenBank database.

Seven distinct genotypes were identified from 18 *I* *T* *S* *1* sequences. The sequence identities varied from 98.4% to 100%, when matched with their own genotypes or with two ITS‐1 reference sequences of *E. tenella* (GenBank accession nos. MZ983633.1 and MN150708.1; Table 1). After aligning seven *I* *T* *S* *‐* *1* genotypes with the reference sequence of *E. tenella* (MN150708.1) and detected six SNPs, which were the result of substitutions in nucleotide positions 53, 83, 107, 146, 215 and 257. There were one transversions (T↔G) and five transitions (four A↔G and one T↔C) in those substitutions (Table 2). The overall nucleotide diversity and genotype diversity were 0.00507 and 0.8235, respectively, among the *I* *T* *S* *‐* *1* sequences of *E. tenella*.

**TABLE 1:** The sequences identities (%) among seven *I* *T* *S* *1* genotypes of *E. tenella* representing 18 samples from Mymensingh using a selected sequence of *E. tenella* from GenBank

**SL no**.	**Sample ID**	**1**	**2**	**3**	**4**	**5**	**6**	**7**	**8**	**9**
1	BDET01 (LC651128)	–								
2	BDET02 (LC651129)	99.2	–							
3	BDET03 (LC651130)	99.6	98.8	–						
4	BDET05 (LC651132)	99.6	99.6	99.2	–					
5	BDET10 (LC651137)	99.2	99.2	98.8	99.6	–				
6	BDET11 (LC651138)	99.2	99.2	98.8	99.6	99.2	–			
7	BDET13 (LC651140)	98.8	98.8	98.4	99.2	98.8	99.6	–		
8	*E. tenella* (MZ983633.1)	100	99.2	99.6	99.6	99.2	99.2	98.8	–	
9	*E. tenella* (MN150708.1)	100	99.2	99.6	99.6	99.2	99.2	98.8	100	–

**TABLE 2:** Nucleotide details and distribution of seven genotypes from 18 samples of *E. tenella* isolated from litter samples of broiler farms

	**Nucleotide position**						
**Genotypes**	53	83	107	146	215	257	**No. of isolates**
MN150708.1	G	A	A	T	G	A	
BDET01 (LC651128)	.	.	.	.	.	.	4
BDET02 (LC651129)	.	G	.	C	.	.	4
BDET03 (LC651130)	.	.	.	.	A	.	1
BDET05 (LC651132)	.	.	.	C	.	.	6
BDET10 (LC651137)	.	.	.	C	.	G	1
BDET11 (LC651138)	T	.	.	C	.	.	1
BDET13 (LC651140)	T	.	G	C	.	.	1
Total							18

### 3.2 Phylogenetic analysis of *E. tenella*

**FIGURE 2:**
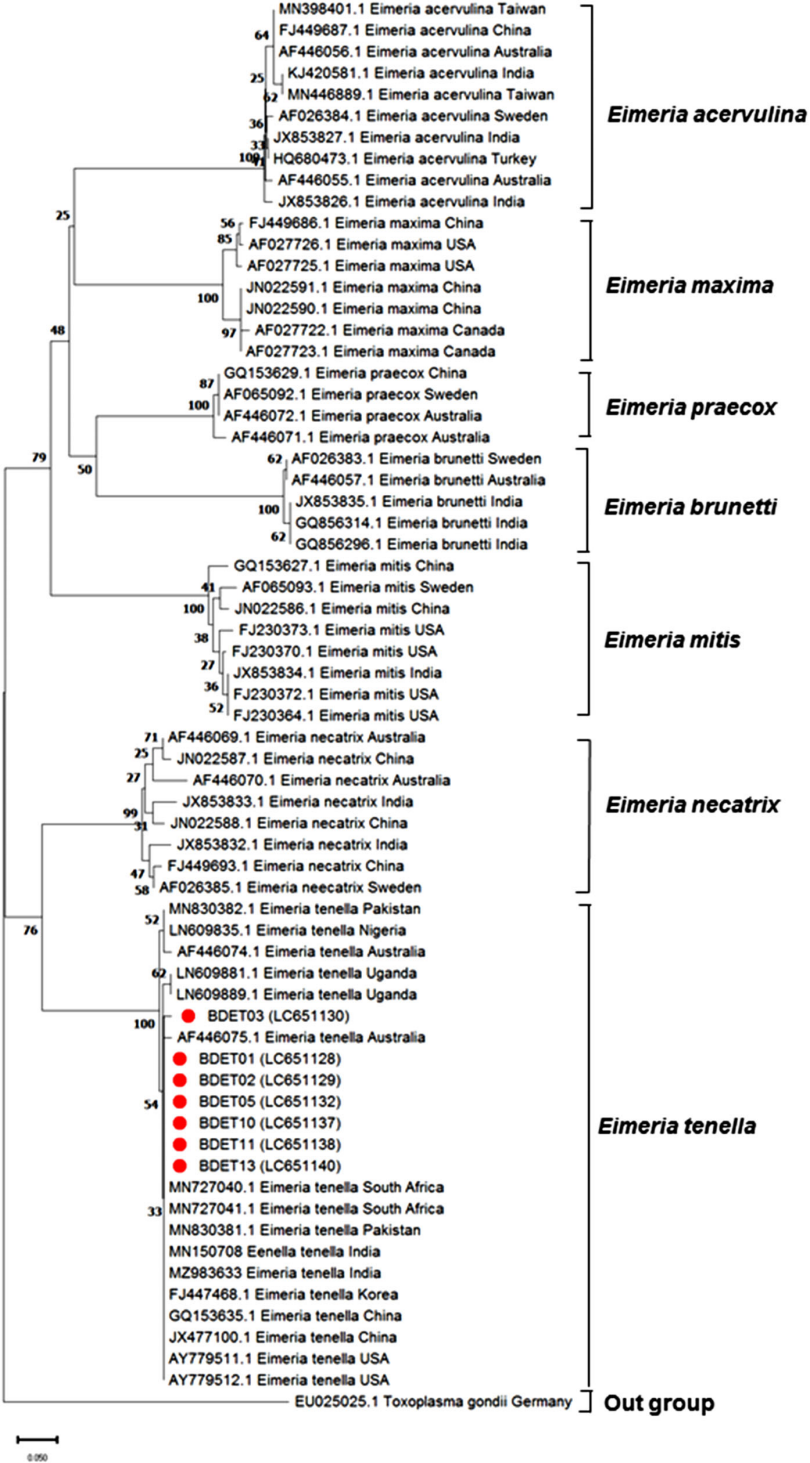
Neighbour‐joining phylogeny of 66 ITS1 gene sequences of *E. tenella*. The analysis involved 66 nucleotide sequences (seven studied genotype sequences and 59 reference sequences, retrieved from the GenBank database). The sequences were aligned and constructed a neighbour‐joining tree. The percentage of replicate trees in which the associated taxa clustered together in the bootstrap test (1000 replicates) are shown next to the branches. The tree is drawn to scale, with branch lengths in the same units as those of the evolutionary distances used to infer the phylogenetic tree. Red bullets indicate studied genotype sequences

We constructed a phylogenetic tree with the seven genotype sequences produced in this study and 59 sequences of different species of *Eimeria* retrieve from NCBI GenBank, represented from Asia, Europe, Africa, Australia and America through the neighbor‐joining method, where *Toxoplasma gondii* (EU025025) acts as an outgroup. The nucleotide sequences of *E. tenella*, *E. necatrix*, *E. acervuline, E. brunetti, E. maxima, E. mitis* and *E. praecox* formed clusters discretely without any distinct boundary in relation to their geographical distribution. The neighbor‐joining tree of *I* *T* *S* *1* sequences of different species of *Eimeria* disclosed that the studied *E. tenella* isolates clustered with previously described *E. tenella* with a strong nodal support value of 100% by bootstrapping. *Eimeria necatrix* and *E. tenella* were positioned in the same sister clade with a bootstrap value of 76%. *Eimeria praecox* and *E. brunetti* also formed sister clades with 50% nodal support by bootstrapping (Figure 2).

## 4 DISCUSSION

Coccidiosis in chickens caused by *Eimeria* spp. are prevalent worldwide including Bangladesh (Alam et al., 2020; Awais et al., 2012; Franco, 1993; Islam et al., 2020; Jenkins et al., 2017). *E. tenella* is the most injurious coccidian agent causing haemorrhagic diarrhoea in chickens and significant loss to the global economy. Susceptibility to coccidiosis is high at the age of 15–50 days, and the estimated morbidity is 50%–70% (Clark et al., 2017; Fornace et al., 2013). To protect commercial and backyard chicken farms, a specific diagnosis of infection is mandatory.

Traditional morphometry of *Eimeria* oocyst is approved as the gold standard. Such diagnoses are also based on oocyst morphology, infection site, pre‐patent period, sporulation time and need intensive labour, expertise and more time (Vrba et al., 2010). Moreover, the size and shapes of oocysts vary due to variation in metabolic activity of parasites or hosts based on infection duration and severity. Therefore, morphometric indices may lead to misdiagnosis (Olufemi et al., 2020). In addition, the involvement of multiple species of coccidiosis is quite common in the field, and close resemblance of morphology and pathological features usually misguide diagnosis leading to neglecting the subclinical form (Kumar et al., 2014). To overcome the difficulties in morphometry‐based diagnosis, molecular diagnostic tools are progressively utilised (Alam et al., 2021; Kawahara et al., 2010; Schnitzler et al., 1999). Variation in the findings between non‐molecular and molecular diagnoses of coccidian parasites has already been reported (Jatau et al., 2016; Olufemi et al., 2020), and greater sensitivity was recorded for PCR‐based identification in detecting even all stages of parasites (Jatau et al., 2016). Several factors can be vital for effective PCR‐based diagnosis. The thick oocyst wall can act as a barrier in generating the desired genomic DNA quality (Fernandez et al., 2003). In addition, PCR inhibitors released from Taq DNA polymerase can influence the PCR reaction (Haug et al., 2007). In this study, we collected litter samples from different broiler farms located in the Mymensingh district in Bangladesh. For the collection of purified DNA templates, we used a commercially available kit (Qiagen QIAamp DNA mini kit) without using any inhibitors for PCR analysis. A successful genetic analysis depends on the accurate choice of a gene. For molecular studies and genetic analyses, nuclear rDNA is extensively used. In the present study, the ITS1 rRNA gene was used as a genetic marker to investigate the phylogenetic analysis and DNA sequence variations of *E. tenella*, compared with other *Eimeria* existing in the GenBank.18S rDNA

Sequences of the *ITS‐1* and *ITS‐2* genomic regions and *1* *8* *S* rDNA, the small subunit rRNA are widely used for the identification, ecological genetic studies and phylogenetic and evolutionary analyses at the taxonomic level of different parasites, including *Eimeria* (Khodakaram‐Tafti et al., 2013; Ogedengbe et al., 2018; Tan et al., 2017). *I* *T* *S* rDNA spacers are generally showing a pattern of homogeneity within species but diverge among species due to concerted evolution (Bower et al., 2008). Due to these characteristics, *I* *T* *S* *1* is frequently used for primer designing by reducing the risk of cross‐reaction with various species. Furthermore, it is considered an important tool for molecular diagnosis and phylogenetic analyses due to its ease of amplifying characteristics, availability of conserved regions, adequate numbers of rRNA clusters, quick evolution rate of variable nuclear loci and sufficient amount of variation to differentiate closely related species (Schnitzler et al., 1999; Schwarz et al., 2009).

Phylogenetic analyses based on *ITS1* rDNA sequences in the present study clearly separated seven species of *Eimeria* such as *E. tenella*, *E. necatrix*, *E. accervulina*, *E. brunetii*, *E. maxima*, *E. mitis* and *E. praecox*. Each cluster is separated by a high bootstrap value (99%–100%). Typically, *E. tenella* and *E. necatrix* in chickens are highly pathogenic and form sister clade in the position irrespective of different geographical location with a bootstrap value of 76%. Similar patterns were shown by Khodakaram‐Tafti et al. (2013), where *E. arloingi*, *E. bovis*, and *E. zuernii*, the most pathogenic *Eimeria* in goats and cattle, clustered in a monophyletic group from other species in spite of biological characteristics.

## 5 CONCLUSION

To the best of our knowledge, this is the first study on phylogenetic analysis of the *ITS1* gene of *E. tenella* from litter samples and provides an evolutionary relationship with *E. tenella* in Bangladesh. In the present study, seven distinct genotypes were identified and six SNPs from 18 *E. tenella* isolates were detected. The data of genetic analysis based on *I* *T* *S* *1* sequence could provide important information for further studies on *E. tenella*, including species conformation and assessments of genetic variations between different geographical locations. Therefore, more extensive investigation with a large number of specimens and with other molecular markers is required to provide more information on the population genetic structure of this parasite. Notably, the findings produced by the present study will be helpful to build up appropriate prevention and control strategies.

## AUTHOR CONTRIBUTIONS

*Conceptualisation, data curation, formal analysis, investigation, methodology, software, visualisation, writing–review and editing*: Anita Rani Dey. *Formal analysis, investigation, methodology, resources, validation, visualisation, writing–original draft*: Sharmin Aqter Rony. *Data curation, investigation, methodology, resources, software, writing–original draft*: Shahnaz Parvin. *Data curation, formal analysis, investigation, methodology, validation, writing–original draft, writing–review and editing*: Shirin Akter.

## CONFLICT OF INTEREST

The authors declare that there is no conflict of interest.

## ETHICS STATEMENT

The authors tried to maintain the highest possible ethical standards in their works without any injury to chickens. The study was approved by the Animal Welfare and Experimentation Ethical Committee of Bangladesh Agricultural University (AWEEC/BAU/2020 (42)).

### PEER REVIEW

The peer review history for this article is available at https://publons.com/publon/10.1002/vms3.799

## Data Availability

The data that support the findings of this study are available from the corresponding author upon reasonable request.
